# Refraction and ocular biometric parameters of preschool children in the Beijing whole childhood eye study: the first-year report

**DOI:** 10.1186/s12886-023-03112-y

**Published:** 2023-09-05

**Authors:** Bidan Zhu, Yunyun Sun, Shana Wang, Xi Qin, Lei Li, Bei Du, Jing Fu, Ruihua Wei

**Affiliations:** 1 Tianjin Key Laboratory of Retinal Functions and Diseases, Tianjin Branch of National Clinical Research Center for Ocular Disease, Eye Institute and School of Optometry, Tianjin Medical University Eye Hospital, 300392 Tianjin, China; 2 Tongzhou Maternal and Child Health Hospital of Beijing, 101101 Beijing, China; 3 Beijing Institute of Ophthalmology, Beijing Tongren Eye Center, Beijing Tongren Hospital, Capital Medical University; Beijing Ophthalmology & Visual Sciences Key Laboratory , 100730 Beijing, China

**Keywords:** Preschool, Refraction, Ocular biometry, cycloplegia, Myopia

## Abstract

**Background:**

Prevention of myopia should begin before school age. However, few population-based cohort studies have investigated refractive status in preschool children with cycloplegia. This study aimed to investigate the post-COVID-19 refraction and ocular biometric parameters of preschool children in Beijing Tongzhou District.

**Methods:**

A population-based cohort study of kindergarten children in Tongzhou District, Beijing, commenced in November 2021. The present study reports data from the first year of the aforementioned population-based study. We selected children aged 3–6 years from nine kindergartens. Biometric parameters, including axial length (AL), anterior chamber depth (ACD), and corneal radius of curvature (CR), were collected before cycloplegia. Cycloplegic refraction was also measured. The spherical equivalent (SE), lens power (LP), and AL-to-CR ratio were calculated. Multiple linear regression analysis was used to analyse the correlation between refraction and ocular biometric parameters.

**Results:**

A total of 1,505 children completed the examination, and a mean SE of 1.24 ± 0.91 D was found. The overall prevalence of myopia was 1.93%. The mean AL, ACD, CR, LP, and AL-to-CR ratio were 22.24 ± 0.70 mm, 3.28 ± 0.26 mm, 7.77 ± 0.26 mm, 26.01 ± 1.56 D, and 2.86 ± 0.07, respectively. Longer AL, deeper ACD, larger AL-to-CR ratio, and lower LP were associated with older age; the CR was not significantly different among different ages. In the multiple linear regression analysis, after adjusting for sex and age, the model that included AL, CR, and LP explained 87% of the SE variation. No differences were observed in the prevalence of myopia or the SE in this particular age range.

**Conclusion:**

The findings of this study suggest that a large proportion of preschool children in Beijing are mildly hyperopic, with a considerably low prevalence of myopia. In preschool children, refractive development was found to present mild hyperopia rather than emmetropia or myopia, a phenomenon that is characteristic of this age range.

## Background

The process by which human eyes transition from hyperopia to emmetropia is often called emmetropization [1]. Both genetic and environmental factors are involved [2–4]. If hyperopia wears off too early in children, they gradually develop myopia [5]. Several studies have demonstrated that the prevalence of myopia among children and adolescents in China and other Asian countries is higher than that elsewhere [6–9]. In China, the high incidence of myopia is a serious public health concern. According to a 2018 national epidemiological survey, the prevalence of myopia in children aged 6 years reached 14.5% without cycloplegia, implying that the prevention and control of myopia must include the preschool age group; otherwise, the myopia prevalence in China may reach 61.8% by 2030 [10].

Few population-based cohort studies have investigated preschool children with cycloplegia. Guo et al. [11] found that in preschool children, the prevalence of myopia is very low, and refraction changed negligibly. However, since the emergence of the COVID-19 pandemic in December 2019, many children have been restricted to their homes for longer periods, outdoor activities have decreased, and the use of electronic screens has increased. These environmental changes have potentially led to alterations in children’s refractive statuses and ocular biometric parameters [12, 13].

To understand the post-COVID-19 refraction and ocular development in preschool children, we initiated a population-based cohort study in Tongzhou District, Beijing. From 2021, the refractive changes and their influencing factors in these preschool children will be obtained via follow-up. This article reports the first-year results of this study.

## Methods

### Study Design and Population

This is a population-based cohort study conducted from November 2021 in kindergartens in Tongzhou District, Beijing. Tongzhou District is a suburban area and serves as the sub-center of Beijing, with economic and population levels falling in the middle range among Beijing’s districts. There are a total of 181 preschools in Tongzhou District, comprising 38 public preschools and 153 private preschools. Using a stratified random cluster sampling method, we selected 9 schools, including 2 public preschools and 7 private preschools, with a total of 1917 participants. Among them, 185 individuals were not permanent residents of Tongzhou District and were excluded from the study due to the difficulty of follow-up. Consequently, invitations to participate were extended to 1732 individuals. Among the invited individuals, 439 potential participants were from public preschools, and 1293 potential participants were from private preschools. The two selected public preschools had 150 and 289 potential participants, respectively, while the seven private preschools had 112, 121, 140, 150, 214, 271, and 285 potential participants each.

The project conformed to the tenets of the Declaration of Helsinki and obtained ethical approval from the Ethics Committee of Beijing Tongren Hospital Affiliated to Capital Medical University (TRECKY2020-152). Before the examination of each kindergarten, we held an online parents’ meeting to explain the purpose and methods of the study, as well as the precautions for cycloplegia. We also obtained the informed consent. A total of 1,515 (87.5%) individuals, with consent forms signed by their parents or legal guardians, participated in the study.

### Eye Examination

The research team consisted entirely of ophthalmologists, optometrists, and eye nurses, each of whom underwent standardised training. The distant vision test was performed using Lea Symbols 3-m Set charts (250,300; Good-Lite, Elgin, IL, USA) to accommodate children’s cognitive ability. Refraction before and after cycloplegia was measured using an autorefractor machine (model RM-800; Topcon, Tokyo, Japan). Each eye was measured three times and averaged. The spherical difference between any two measurements should be < 0.5 D; otherwise, additional measurements were taken. Ocular biometry was performed before cycloplegia using the Lenstar 900 (Haag-Streit, Koeniz, Switzerland). Each eye was measured three times, and each measurement was automatically checked for quality by the instrument. If a measurement was deemed unsatisfactory, additional measurements were conducted. The average of the three tests was calculated as the results. Anterior segment and strabismus examinations were performed by an ophthalmologist.

Cycloplegia was induced by 1% cyclopentolate. Each child was given one drop of 0.5% proparacaine hydrochloride in each eye, followed by two drops of 1.0% cyclopentolate (Cyclogyl; Alcon, Fort Worth, TX, USA) 5 min apart. After 30 min, if the pupil size was ≥ 6 mm and the light reflex was absent, cycloplegia was deemed adequate. Otherwise, an additional drop of cyclopentolate was given, and if the above standard of pupil size and light reflex had not been reached after 15 additional minutes, failure of cycloplegia was recorded.

### Definitions

The spherical equivalent (SE) was equal to spherical diopters (D) + 0.5 × cylindrical diopters (D). Myopia was defined as an SE of ≤ − 0.50 D, emmetropia was defined as 0.50 D < SE < 0.50 D, mild hyperopia was defined as 0.50 D ≤ SE < 2.00 D, and mild-to-high hyperopia was defined as an SE of ≥ + 2.00 D. The corneal radius (CR) was equal to the mean of the flattest and the steepest radius. The axial length (AL)-to-CR ratio was calculated as AL divided by the CR. The lens power (LP) was based on the Bennet–Rabbetts [14] formula, using AL, anterior chamber depth (ACD), SE, and CR.

### Statistical analysis

SAS 9.3 software was used for data processing and analysis. An independent samples t-test was used to compare the ocular characteristics, including SE, AL, ACD, CR, AL-to-CR ratio, and calculated LP, between boys and girls. Trend analysis was used to examine the age-related differences. When normal distribution was not met, the Mann-Whitney test was used for comparisons. Multiple linear regression analysis was used to analyse the correlation between refraction and age, sex, and ocular biometry parameters of the children. There was a strong correlation between the left and right eyes (r = 0.87), and only the right eye was included in the statistical analysis. P < 0.05 was considered statistically significant.

## Results

A total of 1,515 children participated in this study, and 1,505 eventually completed the cycloplegia and ocular biometry examinations. In the public preschools, there were a total of 380 individuals, and the participation rate was 86.6%. In the private preschools, there were 1125 individuals, and the participation rate was 87.0%. There was no statistically significant difference in the participation rates between the two groups (*x*^*2*^ = 0.0084, P = 0.93). Among them, 793 were boys (52.7%). Moreover, 208, 507, 659, and 131 were aged 3, 4, 5, and 6 years, respectively. The average age was 4.97 ± 0.78 years. No statistically significant differences in sex, age, or ethnicity were noted between the included and excluded groups (Table 1).

**Table 1:** Characteristics of children with versus without cycloplegia

	Children with cycloplegia n = 1505	Children without cycloplegia n = 10	P
Sex (boy/girl)	793/712	3/7	0.1481
Age	4.97 ± 0.78	4.85 ± 0.79	0.6162
Ethnic (Han/other)	1208/92	7/2	0.1507

The SE age and sex distributions are presented in Table 2. The average SE was 1.24 ± 0.91 D. The SEs were 1.18 ± 0.94, 1.29 ± 0.88, 1.22 ± 0.93, and 1.19 ± 0.83 D at 3, 4, 5, and 6 years of age, respectively. In the trend analysis, no significant difference was noted in the overall SE or SE by sex. Furthermore, no statistically significant age differences were observed between boys and girls.

**Table 2:** Refractive and biometric characteristics in preschool children in Beijing

Characteristic	3y	4y	5y	6y	Total	P_trend_ value*
Spherical equivalent refraction						
Total	1.18 ± 0.94	1.29 ± 0.88	1.22 ± 0.93	1.19 ± 0.83	1.24 ± 0.91	0.770
Boys	1.22 ± 0.76	1.26 ± 0.86	1.15 ± 1.02	1.17 ± 0.89	1.20 ± 0.92	0.299
Girls	1.12 ± 1.12	1.32 ± 0.90	1.30 ± 0.81	1.22 ± 0.74	1.28 ± 0.89	0.415
P value^#^	0.440	0.391	0.039	0.771	0.088	
Axial length (mm)						
Total	21.96 ± 0.64	22.10 ± 0.67	22.35 ± 0.69	22.60 ± 0.68	22.24 ± 0.70	< 0.001
Boys	22.10 ± 0.62	22.36 ± 0.63	22.60 ± 0.65	22.83 ± 0.61	22.48 ± 0.67	< 0.001
Girls	21.80 ± 0.64	21.84 ± 0.61	22.08 ± 0.63	22.24 ± 0.64	21.97 ± 0.64	< 0.001
P value	< 0.001	< 0.001	< 0.001	< 0.001	< 0.001	
Anterior chamber depth (mm)						
Total	3.20 ± 0.29	3.24 ± 0.25	3.32 ± 0.25	3.40 ± 0.21	3.28 ± 0.26	< 0.001
Boys	3.24 ± 0.32	3.30 ± 0.23	3.38 ± 0.24	3.46 ± 0.20	3.35 ± 0.25	< 0.001
Girls	3.15 ± 0.25	3.17 ± 0.25	3.24 ± 0.24	3.32 ± 0.19	3.21 ± 0.24	< 0.001
P value	0.030	< 0.001	< 0.001	< 0.001	< 0.001	
Corneal radius (mm)						
Total	7.78 ± 0.26	7.76 ± 0.25	7.77 ± 0.26	7.82 ± 0.26	7.77 ± 0.26	0.314
Boys	7.81 ± 0.26	7.83 ± 0.25	7.84 ± 0.26	7.87 ± 0.25	7.84 ± 0.25	0.126
Girls	7.74 ± 0.25	7.70 ± 0.24	7.70 ± 0.25	7.73 ± 0.25	7.71 ± 0.25	0.580
P value	0.054	< 0.001	< 0.001	< 0.002	< 0.001	
AL-to-CR ratio						
Total	2.82 ± 0.06	2.85 ± 0.07	2.88 ± 0.07	2.89 ± 0.07	2.86 ± 0.07	< 0.001
Boys	2.83 ± 0.06	2.86 ± 0.06	2.88 ± 0.07	2.90 ± 0.08	2.87 ± 0.07	< 0.001
Girls	2.82 ± 0.06	2.84 ± 0.07	2.87 ± 0.06	2.88 ± 0.06	2.85 ± 0.07	< 0.001
P value	0.109	0.001	0.002	0.044	< 0.001	
Lens power (D)						
Total	27.33 ± 1.54	26.37 ± 1.46	25.53 ± 1.36	25.07 ± 1.31	26.01 ± 1.56	< 0.001
Boys	26.94 ± 1.49	25.89 ± 1.31	25.16 ± 1.23	24.62 ± 1.19	25.58 ± 1.45	< 0.001
Girls	27.75 ± 1.49	26.87 ± 1.44	25.93 ± 1.37	25.76 ± 1.86	26.49 ± 1.55	< 0.001
*P* value	< 0.001	< 0.001	< 0.001	< 0.001	< 0.001	

Data are presented as mean ± standard deviation

*ANOVA was used to analyse the changing trend of these parameters

# A t-test was used to analyse the differences by sex

AL, axial length; CR, corneal radius of curvature; ANOVA, analysis of variance

**Fig. 1:**
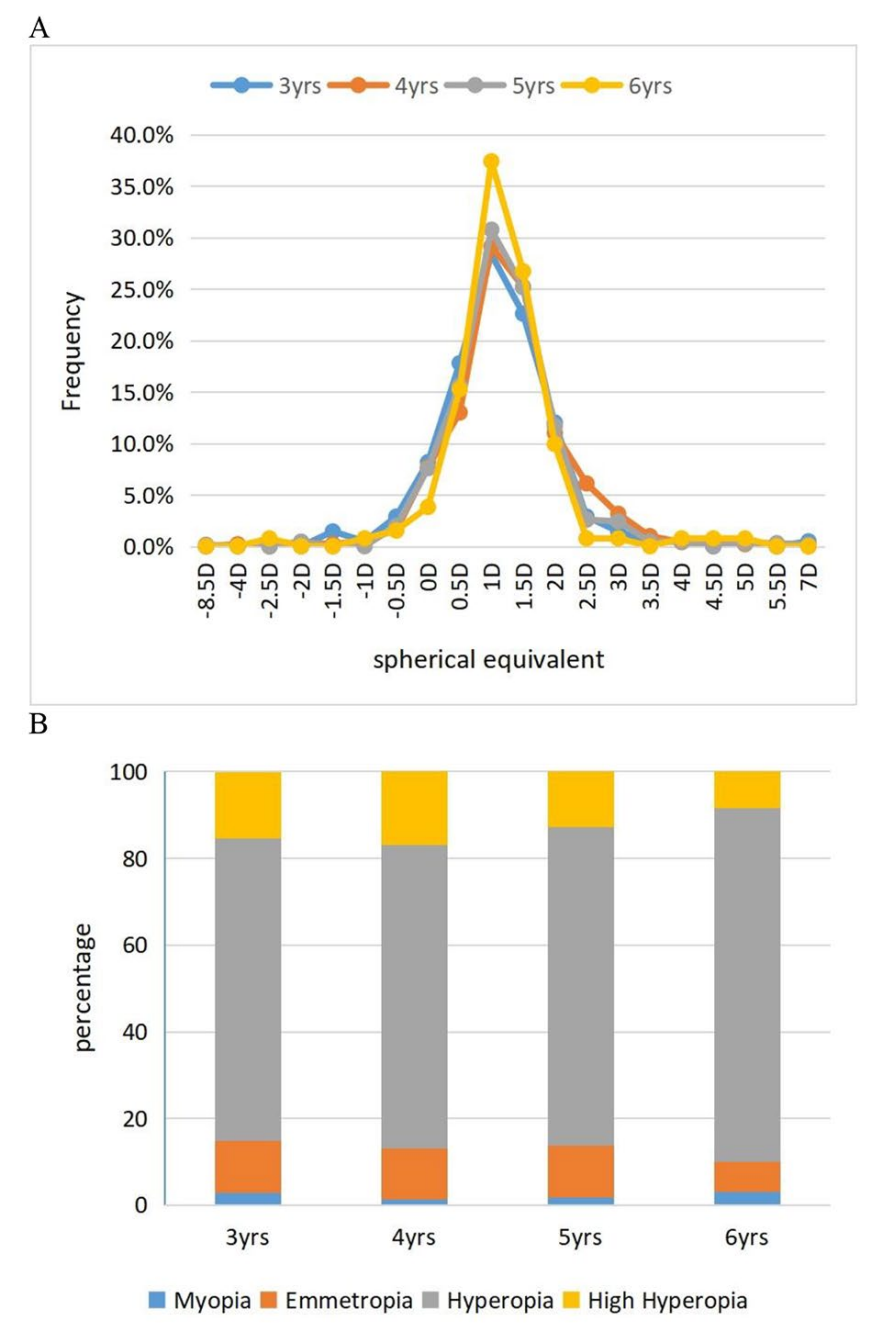
Age-specific distribution of the SE and refractive states. (**A**) Line graph showing age-specific frequency distributions of SE. (**B**) Bar graph showing age-specific distributions of the prevalence of refractive status. SE, spherical equivalent

The refractive-state distribution across various ages is shown in Table 3. In total, 1,304 (86.64%) children were hyperopic. However, at different ages, no significant differences in the proportion of children with hyperopia were noted (P_*trend*_ = 0.404). Mild hyperopia (0.50 ≤ SE < 2.00 D) was the predominant refractive state, and the proportion of children with mild hyperopia increased with age (P_*trend*_ = 0.012). The proportion of children with mild-to-high hyperopia (SE ≥ + 2.00 D) decreased with age (P_*trend*_ = 0.017). The proportion of children with emmetropia and myopia was minimal, and no age differences were noted (P_*trend*_ = 0.358, 0.953). Myopia was rare in this population, with a rate of 3.05% in children aged 6 years. Figure 1 shows the age-specific distribution of the SE and refractive states.

**Table 3:** Distribution of SE in Beijing preschool children by age, no. (%)

SE	3y n = 208	4y n = 507	5y n = 659	6y n = 131	Total n = 1505	*P*_*trend*_ value*
0.50–1.99	145 (69.71)	355 (70.02)	484 (73.44)	107 (81.68)	1091 (72.49)	0.012
≥ 2.00	32 (15.38)	86 (16.96)	84 (12.75)	11 (8.40)	213 (14.15)	0.017
Total (≥ 0.50)	177 (85.10)	441 (86.98)	568 (86.19)	118 (90.08)	1304 (86.64)	0.404
< 0.50, >-0.50	25 (12.02)	59 (11.64)	79 (11.99)	9 (6.87)	172 (11.43)	0.358
≥-0.50	6 (2.88)	7 (1.38)	12 (1.82)	4 (3.05)	29 (1.93)	0.953

*Trend chi-square test was used to analyse the distribution of SE in children aged 3–6 years. SE, spherical equivalent

The age distribution of the ocular biological parameters is shown in Table 2. The AL of preschool children in Beijing was 22.24 ± 0.70 mm (22.48 ± 0.67 mm for boys and 21.97 ± 0.64 mm for girls). Longer AL was associated with older age (P_*trend*_ < 0.001), with measurements increasing from 21.96 ± 0.64 mm at 3 years of age to 22.60 ± 0.68 mm at 6 years of age. Boys had significantly longer ALs than girls aged 3–6 years (P < 0.001). The ACD increased from 3.20 ± 0.29 mm at 3 years of age to 3.40 ± 0.21 mm at 6 years of age (P_*trend*_ < 0.001). ACD was deeper in boys than in girls, with statistically significant differences across all age groups. No statistically significant difference was found in CR among the different age groups during the preschool stage, with an average CR of 7.77 ± 0.26 mm. Except for 3-year-olds, boys exhibited slightly larger CR values than girls, implying that the boys’ corneas were flatter than those of the girls.

The mean AL-to-CR ratio was 2.86 ± 0.07, increasing from 2.82 ± 0.06 to 2.89 ± 0.07 from 3 to 6 years of age (P_*trend*_ < 0.001). Boys had a slightly greater ratio than girls, except for 3-year-olds; nonetheless, the overall difference was negligible. The LP was calculated using the Bennett–Rabbetts formula, with a mean value of 26.01 ± 1.56 D, and demonstrated a significantly decreasing trend from 27.33 ± 1.54 D at 3 years of age to 25.07 ± 1.31 D at 6 years of age (P_*trend*_ < 0.001). Girls had a greater LP than boys at all ages.

Multiple linear regression was used to analyse the relationship between the SE and ocular biological parameters, after adjusting for age and sex (Table 4). Model 1 only included AL for the analysis and explained 21% of the SE variation. When AL increased by 1 mm, SE decreased by − 0.66 D. When AL and CR were included, model 2 explained 51% of the SE variation. AL and SE were negatively correlated (β = − 1.49, P < 0.001), whereas AL and CR were positively correlated (β = 2.86, P < 0.001). When the AL-to-CR ratio was included in the analysis, model 3 explained 40% of the SE variation. The AL-to-CR ratio increased by 0.1, and the corresponding SE changed by − 0.88 D. Model 4 analysed the association between the SE and the calculated LP, AL, and CR values and explained 87% of the SE variation. The SE was negatively correlated with AL (β = − 2.39, P < 0.001) and LP (β = − 0.50, P < 0.001) but positively correlated with the CR (β = 4.10, P < 0.001).

**Table 4:** Linear regression models for spherical equivalent with age, sex, and ocular biometry

	Model 1			Model 2			Model 3			Model 4	
	β	P Value		β	P Value		β	P Value		β	P Value
Age	0.15	< 0.001		0.33	< 0.001		0.24	< 0.001		0.09	< 0.001
Sex*	-0.24	< 0.001		-0.30	< 0.001		-0.07	< 0.001		-0.14	< 0.001
AL (mm)	-0.66	< 0.001		-1.49	< 0.001					-2.39	< 0.001
CR (mm)				2.86	< 0.001					4.10	< 0.001
AL-to-CR ratio							-8.79	< 0.001			
Lens power (D)										-0.50	< 0.001
R^2^	0.21			0.51			0.40			0.87	

*Adjusted for age and sex (boys as reference). AL, axial length; CR corneal radius of curvature

## Discussion

This is a cohort study on cycloplegia refraction and ocular biological parameters in preschool children in Beijing, China. This study reported first-year data and found that a large proportion of preschool children in Beijing were mildly hyperopic. Despite this population’s considerably young age, a certain proportion had myopia; however, the prevalence was < 4%. After COVID-19, the average SE of preschool children in Beijing was 1.24 ± 0.91 D. Longer AL and larger AL-to-CR ratio were associated with older age; the CR was not significantly different between age groups. In the multiple linear regression analysis, the model including AL, CR, and LP explained 87% of the SE variation after adjusting for sex and age.

The myopia incidence in the present study was 1.93%. A kindergarten study in Shenzhen [11] (in southern China) yielded similar results, with a myopia incidence of 1.3%. The myopia incidence among Shanghai kindergartens [15] (on the central coast of China) was 3.7%, which is significantly higher than our result. Among non-Hispanic Whites, the myopia incidence was found to be 1.27% [16]. The myopia incidence of 6-year-old children in the Beijing Pinggu Eye Disease Study was 2.5% [17], while that in our population was 3.05%. Although the incidence of myopia among school-age children in China is higher than that in other ethnic groups [18–21], the difference is not significant in the preschool-age group [11], even after spending more time at home because of COVID-19 [12, 13]. This suggests that the incidence of myopia in Chinese children rises relatively quickly after commencing school. Environmental factors, such as the educational environment and outdoor time, significantly influence the incidence of myopia in Chinese children [3]. This study revealed that the myopia incidence in Beijing is consistent with that in southern China [11, 22] but lower than that in the central coastal area [15]. This may be related to varying educational needs and socioeconomic statuses across different regions.

Hyperopia characterises most refractive states in preschool children. However, upon further categorisation into mild and mild-to-high hyperopia, we found that as age increased, the number of mild-to-high hyperopia cases gradually decreased, that of mild hyperopia cases gradually increased, and that of emmetropia and myopia cases remained stable. This indicates that in the preschool stage, the degree of hyperopia declines and gradually drifts toward mild hyperopia rather than emmetropia or myopia. Typically, preschoolers develop mild hyperopia instead of emmetropia. This is consistent with the results obtained from a one-year follow-up of preschool children in Shanghai [23].

The SE in this study was 1.24 ± 0.91 D, which was slightly lower than that of Shanghai (1.43 ± 0.60D) [23] and Shenzhen (1.37 ± 0.63D) [11] preschool children (Table 5). The SE of non-Hispanic Whites was 1.33 ± 1.15 D in children aged 6–72 months [16] and 1.75 ± 0.97 D in Danish children aged 4.5–7 years [24]. Overall, the average SE in preschoolers worldwide is that of mild hyperopia regardless of ethnicity. The SE in this study was lower than that in most other studies; however, it should be noted that this study was conducted in late 2021 and early 2022, reflecting the refractive statuses of preschool children after COVID-19. No difference in SE was observed across all ages in this study, exhibiting consistency with findings from Guangzhou [22] and Shanghai [15] but not with those from Shenzhen [11], where a downward trend in the mean SE of children aged 3–6 years was recorded.

**Table 5:** Previous studies on refraction and ocular biometric parameters of preschool children

Source	Age	Sample Size (n)	Ethnicity	SE (D)	AL (mm)	CR (mm)
Present study	4.97 ± 0.78 years	1505	Chinese (northern China)	1.24 ± 0.91	22.24 ± 0.70	7.77 ± 0.26
Dirani et al., 2010 [9]	6–72 months	3009	Singaporean Chinese	0.69 ± 1.15		
Lan et al., 2013 [22]	3–6 years	2480	Chinese (southern China)	1.42 ± 0.79		
Wen et al., 2013 [16]	6–72 months	1501	Non-Hispanic white	1.33 ± 1.15		
		1507	Asian children	0.83 ± 1.18		
Zhang et al., 2015 [25]; Lu et al., 2016 [26]	10.0 ± 3.3 (4–18 years)	5913	Chinese (eastern China)	-0.22 ± 2.06	23.45 ± 1.20	7.84 ± 0.27
Guo et al., 2017 [11]	5.0 ± 0.9 years	1133	Chinese (southern China)	1.37 ± 0.63	22.39 ± 0.68	7.79 ± 0.25
Sandfeld et al., 2018 [24]	68 months	445	Caucasians	1.75 ± 0.97		
Zhang et al., 2018 [15]	4.86 ± 0.82 years	2851	Chinese (east China)	1.20 ± 1.05	22.29 ± 0.73	7.83 ± 0.27
Ma et al.,2021 [23]	3–5 years	458	Chinese (east China)	1.43 ± 0.60	22.19 ± 0.69	7.84 ± 0.27

SE, spherical equivalent; AL axial length, CR, corneal radius of curvature.

Our study found that among children aged 3–6 years of age, the older they were, the longer the AL, the deeper the ACD, the greater the AL-to-CR ratio, and the smaller the LP. These findings are consistent with previously reported developmental patterns in school-aged children, marked by axial elongation and LP reductions [26, 27]. The CR was not significantly different between age groups, demonstrating congruency with the results of the Shenzhen kindergarten study [11] and Anyang Children’s Eye Disease Study (6–12 years) [28]. However, previous studies had been inconsistent. Zhang et al. [25] and Scheiman et al. [29] found that the CR had a statistical difference by age. Boys generally have a longer AL and flatter corneas than girls, which corroborates the findings of other studies [11, 30].

The AL-to-CR ratio is an effective indicator of myopia, and the optimal diagnostic cutoff value is reportedly > 2.99 [31]. The AL-to-CR ratio in this study was 2.86 ± 0.07. In our study, we observed that the AL-to-CR ratio increased from 2.82 ± 0.06 to 2.89 ± 0.07 from 3 to 6 years of age. Interestingly, these findings are consistent with the results obtained in the Shenzhen kindergarten eye study. In their study, they reported an AL-to-CR ratio of 2.84 ± 0.06 at 3 years of age, which also became greater with older age, reaching 2.91 ± 0.07 at 6 years of age.

The effectiveness of AL-to-CR ratio in evaluating the SE was statistically superior to that of AL, explaining 40% of the SE variance. Linear regression demonstrated that the offset to myopia was 8.79 D for every one-unit increase in the AL-to-CR ratio. When AL, CR, and LP were included in the predictive calculation model, they explained 87% of the SE variance. AL and LP were negatively correlated with the SE. We observed fluctuations in the mean SE between the ages of 3 and 6. The SE value at age 3 was + 1.18 diopters. Subsequently, there was a gradual increase to + 1.29 diopters at age 4, followed by a subsequent decrease to + 1.19 diopters at age 6. However, in this study, no age difference in the SE was noted, a phenomenon that may be due to the limited age range of 3–6 years; nonetheless, a decrease in LP is known to compensate for the longer AL with increasing age, preventing the development of myopia in children [31–33].

However, our study has certain limitations. First, LP was calculated instead of being measured. Rozema demonstrated that LP calculated using the Bennett–Rabbetts formula was comparable to that measured using the alphabet method in adults with emmetropia and myopia [14]. However, it might not have been suitable for preschoolers with hyperopia in this study. In addition, no records of family history, close work hours, screen use, or time spent outdoors were available to account for the effects of post-COVID-19 changes in eye-use behaviour on refraction and ocular biometric parameters. This information will be published in subsequent analyses. Despite these limitations, our results are consistent with those of the Guangzhou preschool [22] and Shenzhen kindergarten [11] studies.

## Conclusions

We described the first-year data from a cohort study on refraction and ocular biometric parameters in preschool children in Beijing. In this population, the majority had hyperopia, while the proportion of those with myopia was substantially low. With age, mild-to-moderate hyperopia gradually shifted toward mild hyperopia but did not progress to myopia, exhibiting consistency with the results of several studies and potentially elucidating the process of refractive development in children. Changes in the refractive statuses of preschool children in Beijing will be observed upon follow-up.

## Data Availability

The data used and/or analysed during the current study are available from the corresponding author reasonable upon request.

## Abbreviations

ACD: Anterior chamber depth
AL: Axial length
CR: Corneal radius of curvature
LP: Lens power
SE: Spherical equivalent
